# Association between admission serum potassium concentration and the island sign on cranial CT in HICH patients: a cross-sectional study

**DOI:** 10.3389/fneur.2024.1337168

**Published:** 2024-06-04

**Authors:** Yanglingxi Wang, Peng Chen, Yidan Liang, Yongbing Deng, Weiduo Zhou

**Affiliations:** Department of Neurosurgery, Chongqing Emergency Medical Center, Chongqing University Central Hospital, School of Medicine, Chongqing University, Chongqing, China

**Keywords:** hypertensive intracerebral hemorrhage, serum potassium concentration, island sign, non-contrast computed tomography, stroke

## Abstract

**Objective:**

This study aimed to explore the correlation between serum potassium (K^+^) concentration upon admission and the presence of the Island Sign (IS) in cranial CT scans of patients diagnosed with Hypertensive Intracerebral Hemorrhage (HICH), including the potential presence of a non-linear relationship.

**Methods:**

This investigation constituted a single-center cross-sectional study. We systematically gathered comprehensive general clinical characteristics, biological indicators, and imaging data from a cohort of 330 patients diagnosed with HICH. These patients received treatment within the neurosurgery department of Chongqing Emergency Medical Center during the period spanning from July 1, 2018, to July 7, 2023. Our primary objective was to scrutinize the potential connection between serum K^+^ concentration upon admission and the presence of the IS observed in cranial CT scans. To meticulously address this inquiry, we employed logistic regression modeling, thereby meticulously evaluating the correlation aforementioned. Moreover, in order to delve deeper into the intricacies of the relationship, we extended our analysis by employing a smoothed curve-fitting model to meticulously authenticate the potential non-linear interrelation between these two critical variables.

**Results:**

In this investigation, a total of 330 patients diagnosed with HICH were ultimately enrolled, exhibiting an average age of 58.4 ± 13.1 years, comprising 238 (72.1%) males and 92 (27.9%) females. Among these participants, 118 individuals (35.7%) presented with the IS upon admission cranial CT scans, while 212 patients (64.3%) did not exhibit this characteristic. Upon comprehensive multifactorial adjustments, a non-linear association was uncovered between serum K^+^ concentration and the presence of IS. Notably, an inflection point was identified at approximately 3.54 mmol/L for serum K^+^ concentration. Prior to the patient’s serum K^+^ concentration reaching around 3.54 mmol/L upon admission, a discernible trend was observed—every 0.1 mmol/L increment in serum K^+^ concentration was associated with an 8% decrease in the incidence of IS (OR: 0.914, 95% CI: 0.849–0.983, *p* = 0.015).

**Conclusion:**

The findings of this study underscore a negative association between reduced serum K^+^ concentration upon admission and the occurrence of the IS on cranial CT scans among patients diagnosed with hypertensive cerebral hemorrhage. Furthermore, this negative correlation appears to manifest within the realm of a non-linear relationship. This study elucidates the potential significance of serum K^+^ concentration levels among patients with HICH, highlighting the role they play. Moreover, the maintenance of a physiological equilibrium in serum K^+^ concentrations emerges as a conceivable protective factor for individuals within the stroke population.

## Introduction

Hypertensive intracerebral hemorrhage (HICH) stands as a prevailing ailment within the realm of neurosurgery, boasting an annual incidence as elevated as 24.6 per 100,000 individuals (1). At present, non-contrast computed tomography (NCCT) persists as a prevailing choice for both screening and diagnostic measures associated with this condition. In comparison with computed tomography angiography (CTA), it offers a more accessible, expeditious, and widespread alternative (2). The Island Sign (IS), as proposed by Qi Li et al. in their study, presents itself as a novel imaging hallmark unveiled through NCCT. This distinct radiological feature reflects a peculiar multifocal pattern observed within the scans—a distinctive form of irregular hematoma characterized by multiple peripheral microhemorrhages encapsulating a central major hematoma (3).

Hypokalemia, defined as a serum potassium (K^+^) concentration descending below the lower threshold of the normal range (typically <3.5 mmol/L) (4), emerges as a frequent post-cerebral hemorrhage complication. A multitude of investigations have consistently linked hypokalemia to factors such as emesis, fasting, glucose administration, and the utilization of dehydrating agents in the initial phases of cerebral hemorrhage (5). Especially, Jingchuan Liu’s study corroborates that post-cerebral hemorrhage surgical interventions stand as a prominent risk factor for this perturbation in electrolyte levels (6). Nevertheless, scanty information exists regarding the potential direct connection between hypokalemia and HICH. In the same vein, studies examining the correlation between dietary K^+^ intake and stroke risk remain scarce. Notably, a few studies have posited that low dietary K^+^ could potentially emerge as a significant stroke risk factor (7–9).

In recent years, IS has garnered substantial attention as a pivotal radiographic marker discernible in NCCT scans of patients diagnosed with HICH. Numerous scholars have delved into the nuances of this phenomenon, with certain studies asserting its potential to not only gauge immediate hematoma expansion but also to furnish insights into the long-term prognosis of afflicted individuals (10). However, to date, no comprehensive investigation has tackled the relationship between the initial serum K^+^ concentration upon admission of HICH patients and the presence of IS in their cranial CT scans.

Given this gap, we undertook a comprehensive statistical and analytical exploration of clinical and imaging data spanning the past 5 years. Our endeavor aims to unravel the potential association linking admission serum K^+^ concentration with the presence of IS in cranial CT scans, thus contributing to a more nuanced understanding of this intricate relationship.

## Materials and methods

### Research subjects

In this investigation, we systematically amassed data pertaining to the overarching clinical characteristics, initial bioindicators upon admission, and cranial CT images of patients afflicted with HICH. These individuals presented themselves at our medical facility within a span of 2 h subsequent to the manifestation of symptoms associated with cerebral hemorrhage. The research was conducted within the confines of the neurosurgery department at Chongqing Municipal Emergency Medical Center between July 1, 2018, and July 7, 2023.

A comprehensive set of inclusion criteria was meticulously adhered to: (1) all patients underwent cranial CT scans post-hospitalization and were diagnosed with HICH in tandem with their clinical manifestations; (2) all patients underwent pertinent bioindicator examinations subsequent to hospital admission; (3) the temporal interval spanning symptom onset, hospital admission, and completion of imaging and bioindicator assessments did not surpass 2 h for any given patient; (4) all participants were at least 18 years old. Notably, the current cerebral hemorrhage was the first episode in all patients.

**Figure 1:**
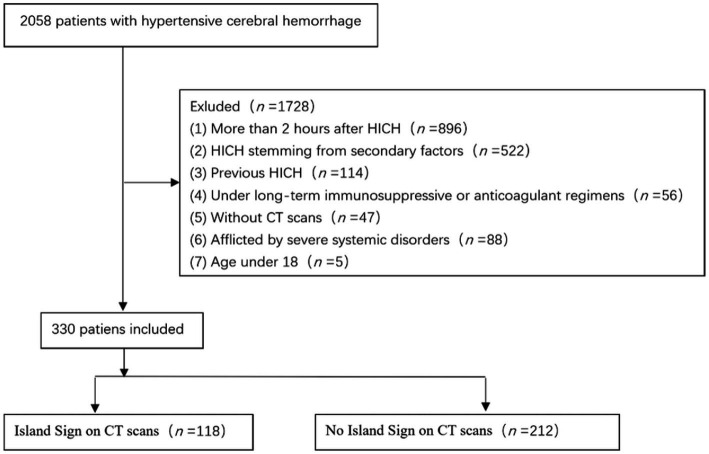
Flow chart of inclusion and exclusion criteria for research subjects. Subsequent to meticulous assessment according to the stipulated inclusion and exclusion criteria, we eventually retained 330 patients for comprehensive analysis from a total of 2,058 individuals afflicted with ICH.

A judicious set of exclusion criteria was concurrently employed: (1) patients with cerebral hemorrhage stemming from secondary factors such as arteriovenous malformations, head trauma, cerebral aneurysms, brain tumors, and cerebral infarctions; (2) patients undergoing long-term immunosuppressive, anticoagulant, or antiplatelet therapy; (3) patients with pre-existing gastrointestinal disorders such as secondary hypertension, primary aldosteronism, Cushing’s syndrome, hyperthyroidism, and conditions causing vomiting and diarrhea; (4) patients with an interval exceeding 2 h between disease onset, hospital admission, and investigative completion; (5) patients with unfinished cranial CT scans or bioindicator tests post-hospital admission due to unrelated reasons; (6) patients with prior history of hemorrhagic strokes; (7) individuals afflicted by severe cardiac, hepatic, pulmonary, renal, or other systemic disorders; (8) patients under 18 years of age. Through meticulous adherence to these criteria, we managed to encompass a total of 2,058 individuals afflicted with hypertensive cerebral hemorrhage. Subsequent to meticulous assessment according to the stipulated inclusion and exclusion criteria, we eventually retained 330 patients for comprehensive analysis (Figure 1).

This subset comprised 238 males and 92 females, spanning an age spectrum from 18 to 91 years (Table 1). Crucially, the research protocol underwent thorough scrutiny and garnered approval from the Ethics Committee of Chongqing Emergency Medical Center.

**Table 1:** Baseline characteristics of participants.

Variables	Total (*n* = 330)	IS		*p* value
		No (*n* = 212)	Yes (*n* = 118)	
Age (years)	58.4 ± 13.1	58.0 ± 13.6	59.0 ± 12.3	0.529
Sex, male, *n* (%)	238 (72.1)	154 (72.6)	84 (71.2)	0.778
Hypertension, *n* (%)	303 (91.8)	194 (91.5)	109 (92.4)	0.784
DM, *n* (%)	45 (13.6)	31 (14.6)	14 (11.9)	0.484
CHD, *n* (%)	35 (10.6)	23 (10.8)	12 (10.2)	0.848
Ischemic stroke, *n* (%)	19 (5.8)	12 (5.7)	7 (5.9)	0.919
CRF, *n* (%)	12 (3.6)	5 (2.4)	7 (5.9)	0.125
Drinker, *n* (%)	64 (19.4)	39 (18.4)	25 (21.2)	0.539
Smoker, *n* (%)	82 (24.8)	55 (25.9)	27 (22.9)	0.537
SBP (mmHg)	175.6 ± 26.4	172.9 ± 26.4	180.7 ± 25.8	0.01
DBP (mmHg)	101.3 ± 16.7	99.9 ± 15.5	103.9 ± 18.4	0.034
Glu (mmol/L)	8.7 ± 2.5	8.7 ± 2.8	8.7 ± 1.8	0.944
GCS score, *n* (%)	10.2 ± 3.2	10.8 ± 3.2	9.2 ± 3.0	<0.001
NIHSS score, *n* (%)	13.5 ± 7.5	11.9 ± 7.4	16.3 ± 6.8	<0.001
^*^ICH score, *n* (%)	2.0 (0.0, 2.8)	1.0 (0.0, 2.0)	2.0 (2.0, 3.0)	<0.001
CT scan				
Cerebral hernia, *n* (%)	94 (28.5)	47 (22.2)	47 (39.8)	<0.001
Volume of cerebral hemorrhage (ml)	23.0 (7.9, 40.0)	14.0 (5.6, 30.4)	33.9 (22.0, 51.7)	<0.001
Midline shift (mm)	3.2 (0.0, 6.0)	2.4 (0.0, 5.0)	4.8 (2.9, 7.5)	<0.001
Thalamic hemorrhage, *n* (%)	125 (37.9)	86 (40.6)	39 (33.1)	0.177
Ventricular hemorrhage, *n* (%)	130 (39.4)	71 (33.5)	59 (50)	0.003
Fourth ventricle hemorrhage, *n* (%)	61 (18.5)	40 (18.9)	21 (17.8)	0.81
Length of the largest section of the fourth ventricle (mm)	6.2 ± 3.0	6.1 ± 3.1	6.3 ± 2.9	0.61
Width of the largest section of the fourth ventricle (mm)	9.9 ± 3.4	9.8 ± 3.5	10.1 ± 3.3	0.41
Laboratory values				
WBC (×10^9^/L)	11.1 ± 4.7	10.3 ± 4.5	12.4 ± 4.8	<0.001
Neu# (×10^9^/L)	9.3 ± 4.7	8.5 ± 4.5	10.7 ± 4.6	<0.001
Neu% (%)	81.2 ± 11.3	79.1 ± 11.6	85.0 ± 9.6	<0.001
Mon# (×10^9^/L)	0.5 ± 0.2	0.5 ± 0.2	0.5 ± 0.2	0.378
Lym# (×10^9^/L)	1.2 ± 0.9	1.3 ± 0.8	1.2 ± 1.0	0.182
HGB (g/L)	138.0 ± 17.9	137.7 ± 17.7	138.4 ± 18.4	0.723
PLT (×10^9^/L)	206.0 ± 72.6	207.8 ± 70.0	202.7 ± 77.2	0.542
PT (s)	13.3 ± 1.9	13.4 ± 2.2	13.2 ± 0.9	0.525
APTT (s)	34.5 ± 10.0	35.0 ± 11.9	33.6 ± 5.2	0.217
INR	1.0 ± 0.2	1.0 ± 0.2	1.0 ± 0.1	0.57
Na^+^ (mmol/L)	139.0 ± 3.7	139.1 ± 3.5	138.9 ± 4.1	0.604
K^+^ (mmol/L)	3.6 ± 0.5	3.6 ± 0.4	3.4 ± 0.5	<0.001

IS, island sign; DM, diabetes; CHD, coronary heart disease; CRF, chronic renal insufficiency; SBP, systolic blood pressure; DBP, diastolic blood pressure; Glu, glucose; GCS, glasgow coma scale; NIHSS, national institutes of health stroke scale; ICH, intracerebral hemorrhage; WBC, white blood count; Neu, neutrophil; Mon, monocyte; Lym, lymphocyte; HGB, hemoglobin; PLT, platelet; PT, prothrombin time; APTT, actived partial thrombolastin time; INR, international normalized ratio; Na^+^, serum natrium; K^+^, serum potassium. ^*^ICH score: the ICH scale, introduced by Hamphill et al. from the University of California in 2001, comprises five criteria: the Glasgow Coma Scale (GCS) score at admission, hematoma volume, patient’s age, presence of intraventricular hemorrhage, and location of the hematoma. Scores range from 0 to 6, with higher scores indicating greater severity of cerebral hemorrhage and poorer prognosis.

### Clinical data

Demographic and general clinical attributes of the 330 HICH patients were meticulously collected in a retrospective manner. These encompassed age, gender, pertinent medical history, blood pressure metrics, blood glucose levels, Glasgow Coma Scale (GCS) score, National Institutes of Health Stroke Scale (NIHSS) score, and Intracerebral Hemorrhage (ICH) score at the point of admission.

Furthermore, comprehensive biomarker data were gathered at admission for all patients. This encompassed White Blood Cells (WBC), Neutrophils (Neu), Neutrophil Count Ratio (Neu%), Monocytes (Mon), Lymphocytes (Lym), Hemoglobin (Hematocrit), Hemoglobin (HGB), Platelet Count (PLT), Prothrombin Time (PT), Activated Partial Thromboplastin Time (APTT), and International Normalized Ratio (INR).

Furthermore, serum sodium (Sodium, Na^+^) and potassium (Potassium, K^+^) concentrations were meticulously recorded. Serum K^+^ concentration gauges the presence of K^+^ ions within the bloodstream, with its normal concentration range delineated as 3.5 to 5.0 mmol/L. This metric is a quantification of the concentration of K^+^ ions within the blood.

### Imaging data

A duo of neuroradiologists, employing a double-blind approach, meticulously evaluated cranial CT scans of the 330 patients. Any disparities that arose were conscientiously deliberated upon and harmonized through consensus. All imaging data were meticulously extracted from the primary images of the NCCT scans captured upon the patients’ admission. The primary scrutiny encompassed factors such as the presence or absence of the combined IS, hematoma volume, magnitude of midline shift, occurrence of concomitant thalamic hemorrhage, existence of conjoined ventricular hemorrhage, presence or absence of combined fourth ventricular hemorrhage, maximal length and width of the largest section of the fourth ventricle and occurrence of combined cerebral herniation.

Estimation of hematoma volume was accomplished using the ABC/2 methodology, as elucidated in a preceding study. This procedure entailed gauging the diameter of the principal hematoma (A) and the perpendicular minor hematoma diameter (B) on the slice harboring the most expansive hematoma area. Augmented by the number of CT levels encompassed by the hematoma multiplied by the thickness of the scanning stratum (C), this enabled the extrapolation of hematoma volume.

For the identification of IS, we adhered to the method outlined in a previous study by Li (3). This entails categorizing IS as follows: (1) presence of ≥3 scattered minuscule hematomas, all individually detached from the principal hematoma; or (2) presence of ≥4 small hematomas, some or all of which might be linked to the major hematoma. The disseminated small hematomas (islands of separation) might adopt a round or oval configuration, distinctly detached from the principal hematoma. Conversely, small hematomas connected to the major hematoma (connected islands) should exhibit a bubbly or budding structure, devoid of lobular attributes (Supplementary Figure S4).

### Statistical analysis

A comprehensive descriptive examination was conducted on the entire cohort of study participants. Categorical variables were succinctly conveyed as proportions (%), while continuous variables were delineated through mean (standard deviation, SD) in the context of normal distribution. Conversely, for distributions exhibiting skewness, they were presented as median (interquartile range, IQR).

In unraveling the link between admission serum K^+^ concentration and IS, logistic regression models were invoked. Results were articulated in the form of odds ratios (ORs) accompanied by 95% confidence intervals (95% CIs), with statistical significance discerned at *p* < 0.05.

To unearth the autonomous connection between serum K^+^ concentration and IS, we resorted to multivariate logistic regression analysis coupled with smoothed curve fitting. Moreover, the affirmation of a non-linear linkage between serum K^+^ concentration and IS was attained through dichotomous linear regression models and smoothed curves. The likelihood ratio test stood employed to compare the one-linear regression model against the dichotomous linear model.

The entirety of the analyses was conducted employing the R 3.3.2 statistical package and Free Statistics software version 1.8 (http://www.R-project.org, R Foundation). Two-tailed tests were conducted, and *p*-values <0.05 were adjudged as indicative of statistical significance.

## Results

### Baseline characteristics of participants

We meticulously scrutinized the baseline data of all 330 patients diagnosed with HICH who were ultimately incorporated into this study (Table 1). The mean age of the entire patient cohort was (58.4 ± 13.1) years, with a notable male predominance, accounting for 238 patients (72.1%). Upon admission, the collective mean systolic blood pressure registered (175.6 ± 26.4) mmHg, while the mean diastolic blood pressure stood at (101.3 ± 16.7) mmHg. Furthermore, the mean GCS score at admission was (10.2 ± 3.2), the mean NIHSS score was (13.5 ± 7.5), and the median ICH score was 2.0 (IQR = 0.0–2.8). The median hematoma volume, as indicated by cranial CT upon admission, measured 23 mL (IQR = 7.9–40 mL), while the median midline shift amounted to 3.2 mm (IQR = 0.0–6.0 mm). Additionally, ventricular hemorrhage was evident in 130 cases (39.4%), and 94 cases (28.5%) displayed manifestations of brain herniation.

Among the cohort of 330 HICH patients, 118 individuals (35.7%) exhibited signs of the Island Sign (IS) upon their admission cranial CT scans, whereas 212 patients (64.3%) did not manifest IS on their cranial CT scans. The collective mean serum K^+^ concentration upon admission was 3.6 ± 0.5 mmol/L. For the subgroup of 118 patients who displayed positive IS on cranial CT, the mean serum K^+^ concentration was 3.4 ± 0.5 mmol/L, whereas the mean serum K^+^ concentration for the remaining IS-negative patients was 3.6 ± 0.4 mmol/L.

### Univariate and multifactorial analysis of admission serum K^+^ and IS

Univariate analysis showed that SBP, DBP, GCS score, NIHSS score, ICH score, cerebral hernia, volume of cerebral hemorrhage, midline shift, ventricular hemorrhage, WBC, Neu, Neu% and serum K^+^ concentration were all statistically significant with the occurrence of IS on cranial CT (*p* < 0.05), which admission serum K^+^ concentration showed *p* = 0.001 in the univariate analysis with IS (OR: 0.38,95% CI: 0.22–0.66) (Supplementary Table S4).

Based on these findings, we proceeded to construct a multifactorial regression model to explore the relationship between admission serum K^+^ concentration and IS (Table 2). In this model, while adjusting for age and gender, we observed that admission serum K^+^ concentration maintained an independent association with the occurrence of IS (OR: 0.90; 95% CI: 0.85–0.96). In Model 2, we expanded our analysis to include a broader range of factors, encompassing SBP, DBP, GCS score, NIHSS score, cerebral hernia, volume of cerebral hemorrhage, fourth ventricle hemorrhage, midline shift, ICH score, WBC, and Neu#. In this context, we still observed a significant association between admission serum K^+^ concentration and IS (OR: 0.92; 95% CI: 0.86–0.98). For Model 3, we conducted a fully adjusted analysis, incorporating the variables from the previous models and introducing additional factors related to ischemic stroke, ventricular hemorrhage, DM, hypertension, and CHD. Even after this comprehensive adjustment, the results persisted: for every 0.1 mmol/L increase in admission serum K^+^ concentration among patients with HICH, there was an 8% reduction in the incidence of IS (OR: 0.92; 95% CI: 0.86–0.98).

**Table 2:** Association between K^+^ and IS using an extended model approach.

	*n*.Total	OR	95%CI	*p*-value
Model1	108	0.9	(0.85–0.96)	0.001
Model2	108	0.92	(0.86–0.98)	0.013
Model3	108	0.92	(0.86–0.98)	0.015

Adjusted covariates: Model1 = Age, Sex. Model2 = Model1 + (SBP, DBP, GCS score, NIHSS score, Cerebral hernia, Volume of cerebral hemorrhage, Fourth ventricle hemorrhage, Midline shift, ICH score, WBC, Neu#). Model3 = Model2 + (Ischemic stroke, Ventricular hemorrhage, DM, Hypertension, CHD).

### Subgroup analysis of admission serum K^+^ concentrations and IS

**Figure 2:**
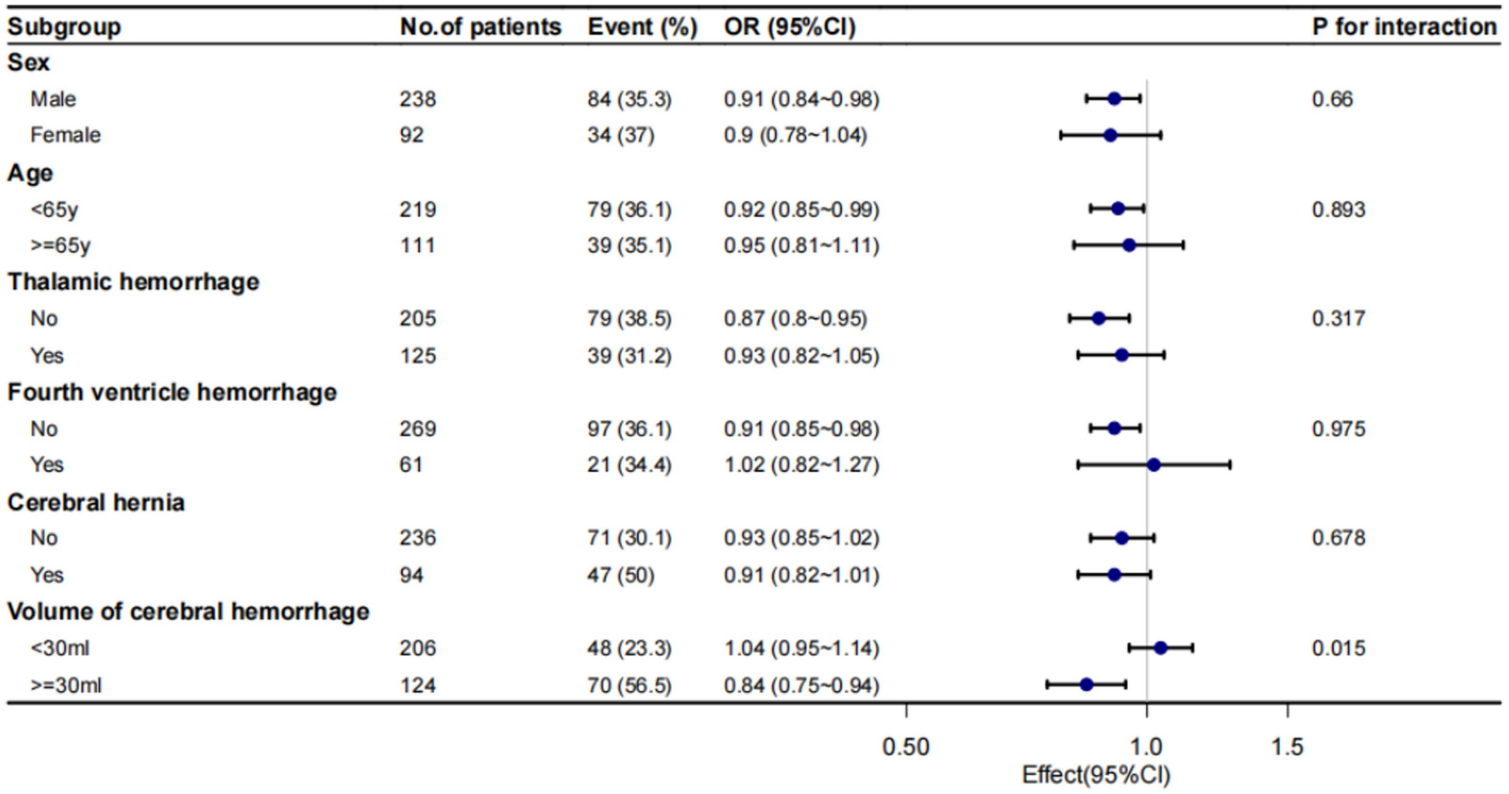
Subgroup analysis of admission serum K^+^ concentrations and IS. Despite performing subgroup analyses based on the confounders, including age, gender, the presence of thalamic hemorrhage, fourth ventricular effusion, cerebral hernia and volume of cerebral hemorrhage.

We conducted a subgroup analysis to investigate the relationship between admission serum K^+^ concentration and IS in all patients (Figure 2). Performing subgroup analyses based on the confounders, including age, gender, the presence of thalamic hemorrhage, fourth ventricular effusion, cerebral hernia and volume of cerebral hemorrhage, a significant interaction was found between serum K^+^ concentration and volume of cerebral hemorrhage (*p* for interaction = 0.015). And, no significant interactions were found in other subgroups (Figure 2).

### Nonlinear relationship between admission serum K^+^ concentration and IS

**Figure 3:**
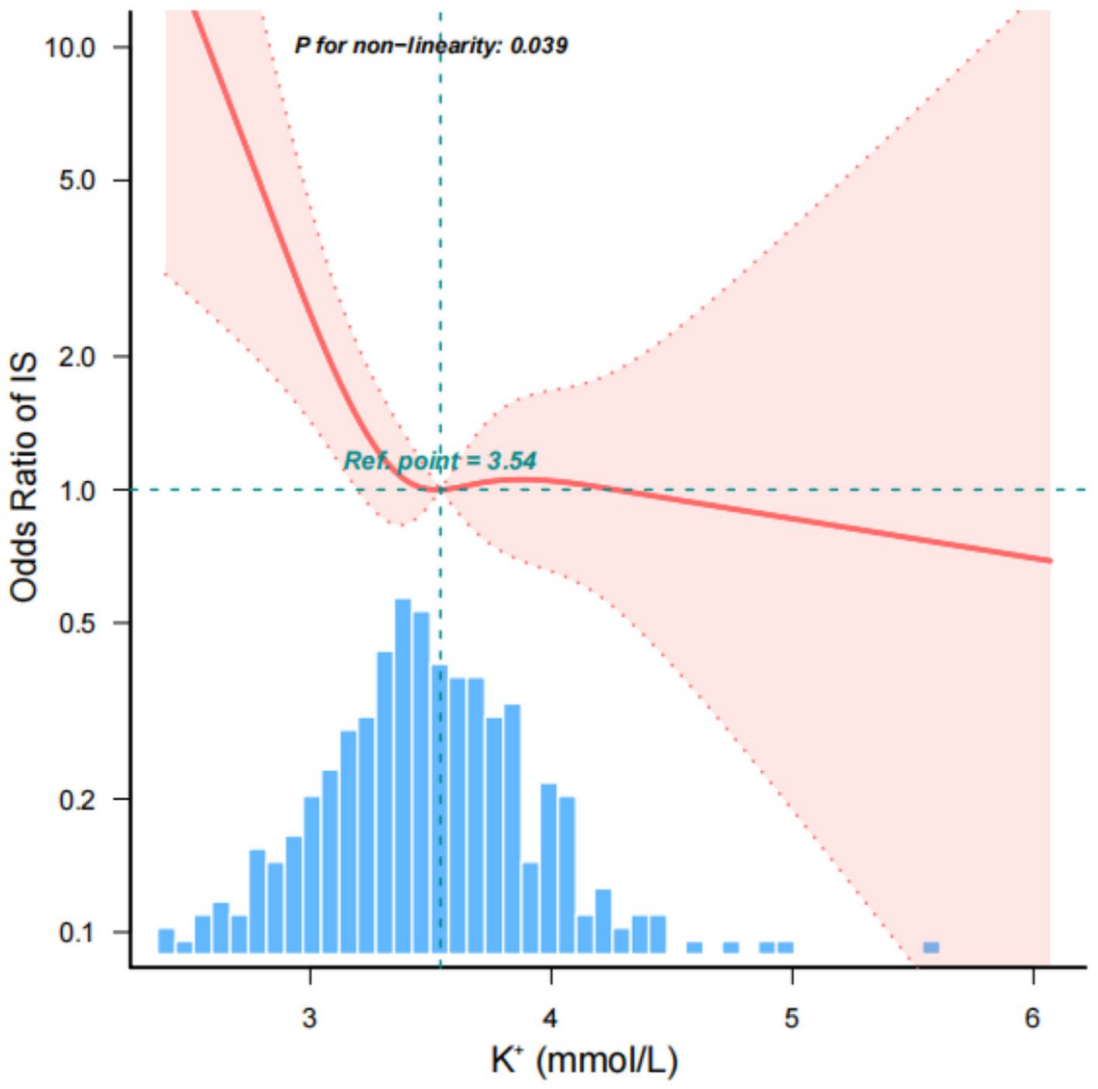
Nonlinear relationship between admission serum K^+^ concentration and IS. This implies that prior to the serum K^+^ concentration reaching around 3.54 mmol/L, the incidence of IS exhibited an 8% decrease for every 0.1 mmol/L increase in its concentration. However, on the right side of the inflection point, the incidence of IS no longer displayed an upward trend with increasing serum K^+^ concentration.

Utilizing a multivariate logistic regression model and smooth curve fitting, we discerned a nonlinear relationship between serum K^+^ concentration and IS (Figure 3). The data were adapted to a segmented multiple logistic regression model, revealing two distinct slopes. In our study, the *p*-value for the nonlinearity test was 0.015 when we encompassed and adjusted for all the factors from the previous multifactor regression analysis in Model 3. Consequently, we employed a two-segmented model to model the relationship between serum K^+^ concentration and IS. Notably, we identified an inflection point at approximately 3.54 mmol/L for serum K^+^ concentration. To the left of this inflection point, the odds ratio (OR) was 0.914 (OR: 0.914, 95% CI: 0.849–0.983, *p* = 0.015). This implies that prior to the serum K^+^ concentration reaching around 3.54 mmol/L, the incidence of IS exhibited an 8% decrease for every 0.1 mmol/L increase in its concentration.

However, on the right side of the inflection point, the incidence of IS no longer displayed an upward trend with increasing serum K^+^ concentration, and there was no statistically significant correlation (Table 3).

**Table 3:** The non-linearity relationship between K^+^ and IS.

	OR	95% CI	*p*-value
K^+^ <3.54 (mmol/L)	0.914	(0.849–0.983)	0.015
Non-linear test			0.03

Adjusted for all covariates in Model3 of Table 2.

## Discussion

In this comprehensive study, we conducted a thorough review of clinical data spanning the past 5 years, encompassing all patients diagnosed with HICH at our medical center. We meticulously collected and analyzed each patient’s general clinical characteristics, admission biometrics, and imaging findings. A primary focus of our investigation was to assess the relationship between serum K^+^ concentration upon admission and the presence of IS signs on cranial CT scans. Our findings, based on a cohort of 330 subjects who met the inclusion criteria, unveiled a negative correlation between serum K^+^ concentration and the occurrence of IS when the concentration was below 3.54 mmol/L. Importantly, this negative correlation exhibited a nonlinear nature. Conversely, when serum K^+^ concentration exceeded this threshold, no such association was observed. In addition, volume of cerebral hemorrhage can modify the association between serum K^+^ concentration and IS. Particularly, the increased serum K^+^ concentration was associated with a significantly lower risk of IS in the ≥30 mL volume of cerebral hemorrhage group.

K^+^, primarily stored intracellularly, traverses cell membranes through active cellular uptake, facilitated by the sodium/potassium adenosine triphosphatase (Na^+^/K^+^-ATPase) pump (11). Among scholars, hypokalemia has garnered recognition as the most prevalent electrolyte disorder in HICH patients, with an incidence rate soaring as high as 50.2% (6). In the aftermath of HICH onset, the body enters a heightened state of stress, instigating the substantial release of hormones such as catecholamines, glucagon, and corticosteroids, accompanied by a marked surge in blood glucose levels. Concurrently, the Na^+^/K^+^-ATPase pump succumbs to regulation by various factors, accelerating the entry of serum K^+^ into hepatocytes and brain cells, consequently leading to a reduction in extracorporeal serum K^+^ concentration (11, 12). It’s noteworthy that many previous studies either included HICH patients within 24 h of disease onset or did not explicitly specify the interval between disease onset and hospital admission for examination. These studies often presented hyperkalemia and hypokalemia as generalized phenomena, with hypokalemia frequently manifesting within a week of HICH onset (6). In our study, strict screening criteria were meticulously applied to ensure that all patients were admitted to the hospital within 2 h of disease onset, with some patients receiving initial clinical assessments in less than 1 h. Consequently, we propose that hypokalemia should not be solely attributed to post-HICH complications.

IS has been described as a specific type of hematoma with a very irregular shape, and Li et al. in their study clearly stated that only small hematomas that resemble air bubbles or buds that are connected to the main hematoma are considered to be “islands” (3). The mechanism of IS is not fully understood, and several studies have suggested that the presence of such isolated high-density areas may represent multifocal active bleeding from multiple ruptured vessels (13), and may have a similar case course to the CTA speckle sign (14). The majority of studies have focused on the ability of IS to predict secondary expansion of hematomas and to assess the poor prognosis of HICH (15, 16), while few reports have been made on other aspects of the condition. Zhang et al. in their study noted that neutrophil/lymphocyte (Neutrophil to lymphocyte ratio, NLR) can be used as an independent marker reflecting IS in patients with HICH, and the significance behind it may be related to the inflammatory cascade response after hemorrhage as well as the systemic immune status. He also noted that although systemic neutrophils are involved in the pathological progression of active hemorrhage, hematoma reexpansion may be a slower pathological evolution compared to the appellate mechanism, and thus elevated NLR may independently predict the presence of insular signs but not hematoma expansion (17).

In this study, we selected a group of patients with ultra-early HICH from onset to consultation and saw from them that hypokalemia at admission may have increased the incidence of IS, and this correlation existed as a nonlinear relationship with a serum K^+^ concentration of 3.54 mmol/L as the inflection point. Our analysis of clinical practice and relevant literature revealed the following insights: firstly, following an HICH event, blood constituents such as erythrocytes, leukocytes, and macrophages, along with plasma proteins like thrombin, promptly infiltrate the brain parenchyma (18), initiating a cascade of early systemic inflammatory, immune, and coagulation responses. These pathophysiological changes constitute the primary mechanisms underlying the onset of the current IS. Furthermore, imbalances in electrolyte levels (e.g., decreased concentration of serum Ca^+^, Mg^+^, K^+^, and P^+^) upon the patient’s hospital admission can impact the aforementioned pathophysiological processes (19). Secondly, as previously noted, the occurrence of IS signifies the presence of multiple ruptured vessels within the skull, with the active hemorrhage being influenced by the degree of hypertension and cerebrovascular conditions, in addition to the underlying pathophysiological mechanism (3). Furthermore, serum K^+^ concentration supplementation typically leads to a reduction in blood pressure. Even a slight, long-term decrease in blood pressure (as little as 5 mmHg) may result in a swifter, smoother, and more effective lowering of blood pressure in patients with HICH post-admission to the hospital (20). This antihypertensive effect could be attributed to decreased renal vascular resistance and increased glomerular filtration rate (21). Lastly, maintaining elevated levels of serum K^+^ also contributes to preserving endothelial cell integrity in the presence of hypertension. This helps render cerebral arteries more resilient and less susceptible to damage under higher arterial pressures, thereby reducing the likelihood of active bleeding from ruptured vessels following HICH (22, 23). Although there is currently a lack of explicit studies examining the definitive evidence linking blood potassium levels to IS, the proposed pathophysiological mechanisms lend support to our findings to some extent.

In subgroup analysis, we observed a significant association between serum K^+^ concentration and IS specifically in the group with a cerebral hemorrhage volume of ≥30 mL. We postulated that this association could be linked to risk factors contributing to disease severity. Patients with high-volume cerebral hemorrhages inherently present with more challenging hypertension control, poorer coagulation status, a higher prevalence of underlying diseases (particularly cardiovascular conditions), and a more pronounced inflammatory and immune response. All of these factors are closely intertwined with the mechanisms underlying IS occurrence as discussed earlier. Consequently, the incidence of IS may have been elevated in this subgroup of patients presenting with higher serum K^+^ concentration upon admission compared to those with low-volume cerebral hemorrhages (24).

While specific electrolyte imbalances can indeed disrupt the coagulation cascade (25) and directly influence the vasoactive response to some extent (26), it’s crucial to recognize that serum K^+^ concentration are intricately linked with cardiovascular and urinary systems as well. Higher serum K^+^ levels may exert a protective effect against the atherosclerotic process and thrombosis. This is achieved through the reduction of free radical formation, inhibition of smooth muscle proliferation, attenuation of arterial thrombosis, and suppression of platelet aggregation (22, 23), particularly in patients with arrhythmias and underlying cardiac conditions (27). The enduring antihypertensive effect and preservation of renal function were also discussed earlier (21). Additionally, studies by Smith NL and Levine SR et al. have indicated that low serum K^+^ concentration could potentially increase the risk of hemorrhagic stroke, while elevated serum K^+^ concentration within the physiological range might confer protection against stroke (28, 29). So we think that maintaining appropriate and balanced blood K^+^ levels could potentially reduce the risk of stroke and multifocal active hemorrhage. In China, there is a dearth of research focused on the dietary intake of specific elements, particularly among middle-aged and elderly individuals with high-risk factors for stroke, such as hypertension. Based on our clinical observations, we noticed that some patients had irregular dietary habits, an uneven nutritional profile, and excessive consumption of processed foods before the onset of their illnesses. With the advancement of industrialization and technology, food processing techniques have improved significantly, introducing a plethora of processed foods into people’s lives. This has disrupted the balance of nutrient intake, particularly concerning sodium and potassium (30). Concurrently, the progress of civilization has led to a reduction in dietary K^+^ intake (21). Consequently, increasing K^+^ intake within acceptable limits may prove beneficial for individuals with multiple stroke risk factors and those who have already experienced a stroke. We also propose that in patients in the hyperacute phase of hypertensive cerebral hemorrhage, it might be reasonable to correct electrolyte imbalances promptly. However, it is crucial to underline that more substantial evidence is required. Our goal is to maintain a physiologic balance of serum K^+^ concentration following hospital admission, without advocating for artificial or indiscriminate increases in electrolyte levels.

### Limitations

Our study does come with several limitations. Firstly, despite our efforts to maximize the sample size by implementing strict inclusion and exclusion criteria and controlling for major potential confounders, there may still be unmeasured and unknown factors that elude our analysis. This is a common challenge inherent to retrospective studies. Secondly, the cross-sectional nature of our study presents a limitation, as it hinders the establishment of causal relationships. Thus, the precise causal link between serum K^+^ concentration and IS in HICH patients during the hyperacute phase remains not entirely elucidated. Future prospective investigations are warranted to corroborate our findings. Additionally, research on the relationship between serum K^+^ concentration and stroke, particularly within the Chinese population, is still relatively scarce in both animal and epidemiological studies. Therefore, there is a need for further exploration in both basic and clinical research in this domain. Lastly, while our data encompass patients with ICH across various ages, genders, and severity levels, spanning emergency, outpatient, and prehospital settings, as well as multiple provinces, they may not entirely reflect the entire population. In our ongoing research, we aspire to collaborate with additional medical institutions to broaden our dataset and extend our scientific inquiries.

## Conclusion

In patients with HICH, our study suggests that admission serum K^+^ concentration exhibits a negative correlation with the presence of IS on cranial CT. This correlation appears to follow a nonlinear pattern. Maintaining a physiological balance in serum K^+^ concentration may serve as a protective factor for individuals at risk of stroke.

## Data availability statement

The raw data supporting the conclusions of this article will be made available by the authors, without undue reservation.

## Ethics statement

The studies involving humans were approved by the Ethics Committee of Chongqing Emergency Medical Center. The studies were conducted in accordance with the local legislation and institutional requirements. Written informed consent for participation was not required from the participants or the participants’ legal guardians/next of kin.

## Author contributions

YW: Conceptualization, Data curation, Formal analysis, Methodology, Validation, Writing – original draft, Writing – review & editing. PC: Conceptualization, Data curation, Formal analysis, Supervision, Validation, Writing – review & editing. YL: Conceptualization, Data curation, Investigation, Writing – review & editing. YD: Funding acquisition, Investigation, Resources, Writing – review & editing. WZ: Methodology, Project administration, Validation, Visualization, Writing – review & editing.
