# Supplementary material for: Association between admission serum potassium concentration and the island sign on cranial CT in HICH patients: a cross-sectional study
Source: Front Neurol. 2024 Jun 4;15:1337168. doi: 10.3389/fneur.2024.1337168 (PMC11184062; doi:10.3389/fneur.2024.1337168)
Supplement: Supplementary file 1 [file Table_1.docx]

**Supplementary Figure 1 Island sign on Cranial CT in HICH Patients**


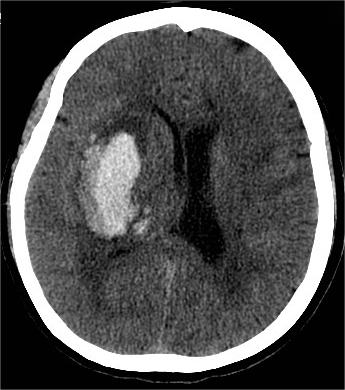


**Figure 1-1** The patient presented to the hospital with a complaint of 'left-sided limb weakness, associated with a 1.5-hour decrease in consciousness.' The GCS score upon admission was 10 (E2V3M5), and the muscle strength in the left-sided limb was categorized as Grade I. Initial serum K^+^ concentration was measured at 3.2 mmol/L, and cranial CT scan revealed clear evidence of IS.


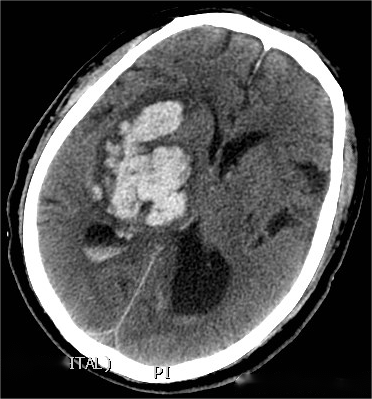


**Figure 1-2** The patient's hospital admission was prompted by a documented '2-hour loss of consciousness.' The patient's GCS score at admission was 8 (E1V2M5), and no response in the left-sided limb was elicited upon painful stimulation. Initial serum K+ concentration measured 3.3 mmol/L. Cranial CT scan revealed basal ganglia hemorrhage accompanied by mass effect and features suggestive of IS.
